# Surgical management of burns in functional areas: a 5-year retrospective study

**DOI:** 10.25122/jml-2025-0077

**Published:** 2025-04

**Authors:** Matei Iordache, Eliza-Maria Bordeanu-Diaconescu, Andreea Grosu-Bularda, Mihaela-Cristina Andrei, Adrian Frunza, Sabina Grama, Raducu Costache, Tiberiu-Paul Neagu, Ioan Lascar, Cristian-Sorin Hariga

**Affiliations:** 1 Department of Plastic Surgery and Reconstructive Microsurgery, Carol Davila University of Medicine and Pharmacy, Bucharest, Romania; 2 Burn Centre, Emergency Clinical Hospital of Bucharest, Bucharest, Romania

**Keywords:** burns, functional area, head and neck, hands, joints

## Abstract

Burns represent one of the most complex types of trauma that can occur in the human body and, as such, remain a subject of constant debate in the medical world. Globally, burns account for approximately 180,000 deaths annually, with the vast majority (95%) occurring in low- and middle-income countries. Severe burns, which involve more than 20% of the total body surface area (TBSA), lead to high mortality and morbidity rates, more so when they affect some of the critical areas such as the face or the hands, feet, and perineum. Each region has its characteristics and challenges that arise from injury to these parts, and thus, each anatomical section should be individually studied to help determine how to prioritize one over the others. In this way, a guideline could be developed to treat the burned patient effectively. A key issue is where to begin—should functional areas be prioritized over others, or should the reverse approach be taken? Current literature has failed to establish a clear algorithm for the optimal management of these patients. This article takes into account the latest recommendations and compares them with our experience and results.

## INTRODUCTION

Burns represent one of the most complex types of trauma that can affect the human body and, as such, remain a subject of ongoing debate in the medical world [1]. They have been considered a significant medical issue since antiquity, with early documentation found in ancient Egypt, China, and Greece [2,3]. In modern times, burns are responsible for approximately 180,000 deaths annually, with 95% of these occurring in low- and middle-income countries, according to the World Health Organization (WHO). Most incidents are reported in domestic settings, with over 1 million people suffering burns each year in India and more than 410,000 injuries occurring annually in the United States. The large number of victims and the considerable economic burden underscore the importance of prevention efforts [4,5]. Severe burns, which involve more than 20% of the total body surface area (TBSA), lead to high mortality and morbidity rates, more so when they affect some of the critical areas such as the face or the hands, feet, and perineum [6-8].

In recent years, there has been a notable decline in both the incidence and mortality associated with burn injuries, although the figures remain concerning. In addition, the length of hospital stay (LOS) per percentage of TBSA has decreased from 1.5–3 days to 0.5–1.4 days. Over the past decade, several studies published in Europe have reported reductions in burn incidence, mortality, severity, and LOS [9].

The American Burn Association (ABA) has elaborated guidelines for burn treatment and referral criteria. Burns involving functional areas, such as the face, hands, feet, perineum, genitalia, or major joints, are important criteria for patient referral. Although their importance has long been stated, the necessity to further study the issue arises, as these regions are commonly affected, and their impact on mortality and morbidity is major [10,11].

Each anatomical area has its functional characteristics and complications, necessitating individualized evaluation. Therefore, it is important to assess each region to determine treatment priorities. This raises an essential clinical question: Should treatment begin with functional areas, or should other regions be addressed first? To date, the literature has not provided a clear, standardized algorithm for prioritizing treatment in patients with extensive burns. This article examines current recommendations in the context of our clinical experience and outcomes, aiming to contribute to the ongoing effort to optimize burn management strategies..

## MATERIAL AND METHODS

This study is a retrospective analysis of 481 patients admitted to the Burn Unit of the Clinical Emergency Hospital of Bucharest between January 1, 2019, and December 31, 2023. Patients transferred from our unit or those with insufficient medical data were excluded from the analysis.

The following demographic and clinical variables were collected: age, gender, percentage of total body surface area (%TBSA) burned, burn degree (including presence of third-degree burns), mechanism of injury, presence of inhalation injury, LOS, and surgical procedures performed. An important component in assessing patient prognosis was calculating the Abbreviated Burn Severity Index (ABSI) score, which is widely used to estimate burn-related mortality risk.

Statistical analysis was performed using Microsoft Excel and IBM SPSS Statistics version 24. Chi-square (χ^2^) tests were applied, and results were reported with corresponding χ^2^ and P values, as well as frequencies and 95% confidence intervals where appropriate.

## RESULTS

A total of 515 patients were admitted for burn injuries during the 5-year study period. Of these, 34 were excluded due to insufficient data or inter-hospital transfer, leaving 481 patients eligible for analysis based on demographic and clinical characteristics.

Patient ages ranged from 18 to 96 years, with a mean age of 55 (54.49 ± 18.61), a median of 53 years, and a symmetrical distribution (skewness = 0.08, kurtosis = −0.86), although variability was high. A significantly greater proportion of patients were men (*n* = 306; 63.6%) compared to women (*n* = 175; 36.4%), with this difference being statistically significant (|^2^_(df=1)_ = 35.68; *P* < 0.001). Regarding place of origin, 248 patients (51.6%) were from rural areas and 233 (48.4%) from urban areas, with no statistically significant difference between the two groups (|^2^_(df=1)_ = 0.24; *P* = 0.624).

A large proportion of patients presented with superficial partial-thickness burns (IIA, *n* = 341), with deep partial-thickness (IIB) burns noted in 408 cases and full-thickness (III) burns in 269 cases (Table 1). Superficial partial burns and deep partial-thickness burns co-occurred in 72.8% of patients (|^2^_(df=1)_ = 4.70; *P* = 0.030), while full-thickness burns were associated with superficial burns in 50.2% of cases (|^2^_(df=1)_ = 126.84; *P* < 0.001). Further analyzing the more severe burns, it was noted that 59.3% of deep partial burns also had full-thickness burns, while of the patients with third-degree burns, 90% also had deep partial burns, being statistically significant (|^2^_(df=1)_ = 12.52; *P* < 0.001).

**Table 1:** Frequency of each burn degree of the skin and of the inhalation injuries

Burn degree	*n*	Percentage (%)	Chi-Square Test of Proportion		
			X^2^	df	*P*
IIA	341	70.9	83.99	1	<0.001
IIB	408	84.8	233.32	1	<0.001
III	269	55.9	6.76	1	<0.010
Inhalation injury	138	28.7	87.37	1	<0.001

The average TBSA burned was 27.5%, ranging from 1% to 99%. One-quarter of patients had burns, covering more than 40% of TBSA. The mean hospital length of stay was 24 days (23.42 ± 23.47), ranging from 1 to 131 days, with 25% of patients hospitalized for over 30 days. Most patients (73.5%) were admitted to the critical care unit, while only 26.5% had less severe injuries.

The most commonly affected functional areas were the cephalic extremity and the hands (Table 2). Among cephalic burns, 98.1% involved the face, and 53.9% involved the cervical region. Joint involvement included shoulders (41%), elbows (62.9%), hands (63.6%), hips (27.1%), knees (53.3%), and ankles (30.1%). Notable associations included cephalic burns with hand burns in 77.5% of cases and with shoulder (87.2%) and elbow burns (84.1%).

**Table 2:** The distribution of the main functional areas with corresponding numbers and frequency

Affected region	*n*	Percentage (%)	Chi-Square Test of Proportion		
			X^2^	df	*P*
Cephalic extremity	319	66.3	51.25	1	<0.001
Hand	306	63.6	35.68	1	<0.001
Ankle	145	30.1	75.84	1	<0.001
Joints	229	47.6	1.10	1	0.294
Perineum	86	17.9	198.51	1	<0.001

**Figure 1:**
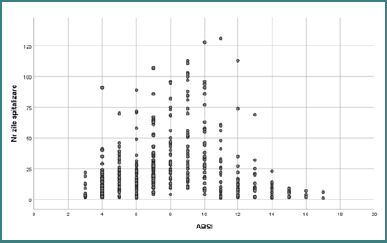
The graphic relationship between the ABSI score and LOS

Out of 481 patients, 137 died (28.5%). The mean ABSI score was 7.31 ± 3.09, with 50% of patients scoring ≤7 and 25% scoring ≥9 (Figure 1). Survivors had a mean ABSI of 6.11 ± 2.00, compared to 11.01 ± 2.54 in deceased patients.

We investigated the statistical correlations between various factors and increased mortality, analyzing sex, age, place of origin, presence of inhalation injury, burn depth, %TBSA affected, anatomical regions involved, and the ABSI score. Age emerged as a strong positive predictor of mortality, with patients over 55 years old having an 11-fold higher risk of death compared to those younger than 55 (B = 2.42; SE = 0.42; Wald_(df=1)_ = 32.61; *P* < 0.001). The presence of inhalation injury was associated with a 9-fold increase in mortality (B = 2.17; SE = 0.42; Wald_(df=1)_ = 26.31; *P* < 0.001). ABSI score was also a statistically significant predictor, with each unit increase corresponding to a 3-fold rise in mortality risk (B = 0.95; SE = 0.22; Wald_(df=1)_ = 19.23; *P* < 0.001).

An analysis of the correlation between burns affecting functional anatomical areas and increased mortality revealed several notable findings. Facial burns were associated with a threefold increase in the risk of death (B = 1.00; SE = 0.54; Wald_(df=1)_ = 3.43; *P* = 0.064). Among all regions assessed, perineal burns demonstrated the strongest association with mortality, corresponding to a sixfold increase in risk (B = 1.81; SE = 0.50; Wald_(df=1)_ = 13.21; *P* < 0.001).

Of the 481 patients included in the study, 266 (55.3%) required surgical intervention. Among these, 63.9% underwent a single operation, while 93.2% required up to three surgical procedures. The most frequently performed procedure was skin grafting, conducted in 188 patients (37.4%). Emergency fasciotomy for compartment syndrome decompression was the second most common intervention, performed in 103 patients (20.5%). Additional procedures included amputations in 16 patients (3.2%), excision and suturing of burn lesions in 19 patients (3.8%), flap reconstruction in 17 cases (3.4%), and tracheostomy in 19 patients (3.8%).

## DISCUSSION

The clinical importance of functional anatomical areas in burn injuries has long been recognized, yet the need for more comprehensive data and standardized treatment protocols in cases of major burns remains pressing. A step-by-step analysis of these regions is necessary to derive the relevant conclusions further. Each anatomical region presents unique features, functional significance, and clinical implications. In many cases, the co-involvement of multiple functional areas can worsen the patient’s overall prognosis, contributing to increased morbidity and mortality. These complex cases pose significant challenges to plastic surgeons, the intensive care doctor, and the broader multidisciplinary care team managing critically burned patients.

In our cohort, we analyzed the demographic characteristics of patients in conjunction with the involvement of specific functional regions to better understand their contribution to clinical outcomes in major burns. The mean patient age was 55 years (54.49 ± 18.61; 95% CI, 52.83–56.16), which is notably higher than figures reported in other studies, where mean ages were 41.4 ± 24.7 and 30 years, respectively [9,12]. Most injuries occurred in domestic settings rather than in occupational environments. The urban-rural distribution of patients was relatively even, with 48.4% originating from urban areas and 51.6% from rural areas. This distribution is likely influenced by our status as a national referral center, receiving patients from across the country. Age was found to be a significant predictor of mortality. Patients older than 55 years had an 11-fold higher risk of death compared to younger individuals (B = 2.42; SE = 0.42; Wald_(df=1)_ = 32.61; *P* < 0.001; OR = 11.28; 95% CI, 4.91–25.90). Gender distribution in our study was consistent with previous findings, such as those reported by Abarca *et al*. in their analysis of 2,651 patients over eight years [9]. We observed a predominance of male patients (63.6%), comparable to the 61.9% reported in their study.

The most prevalent mechanism of injury in our cohort was flame burns, documented in 263 cases (54.7%), which is higher than figures reported in other studies [9]. Other frequently observed mechanisms included scald injuries (*n* = 95; 19.8%) and flash-related burns (*n* = 75; 15.6%) (Table 3). Less commonly, patients presented with electrical injuries (*n* = 17; 3.5%), contact burns (*n* = 16; 3.3%), and chemical burns (*n* = 15; 3.0%).

**Table 3:** Mechanisms of burns and their frequency

Burn mechanisms	Frequency	Percent (%)
Chemical	15	3.0
Contact	16	3.3
Electrocution	17	3.5
Flash	75	15.6
Flame	263	54.7
Scald	95	19.8

It is well established that both the size and depth of burn injuries significantly influence mortality and long-term functional outcomes. Of these two factors, burn size plays a central role in medical decisions beginning from the prehospital stage until rehabilitation as well as aftercare, guiding estimation of fluid resuscitation volumes and nutritional needs, helping in predicting the length of stay, as well as being an important factor in calculating severity scores. A longstanding clinical consensus has established the importance of describing burn size as a percentage of the TBSA affected [13]. As a national referral center, our facility admitted patients with higher severity profiles, with 73.5% requiring critical care unit admission. The average TBSA burned in our cohort was 27.5%, with values ranging from 1% to 99% in the most severe cases. Notably, 25% of patients sustained burns covering more than 40% of their TBSA. In contrast, other studies have reported significantly lower mean TBSA values, such as 8.3%, and correspondingly shorter ICU stays [14]. Burn severity in our cohort is further evidenced by the high incidence of full-thickness (third-degree) burns, affecting 55.9% of patients, more than double the rates reported in other publications (23.4% and 27.7%) [6,9]. Furthermore, some frequent associations could be described: 59.3% of patients with deep partial-thickness burns also had full-thickness burns, while 90% of those with full-thickness burns also had deep partial thickness burns, with a significant statistical correlation (|^2^_(df=1)_ = 12.52; *P* < 0.001), thus importantly increasing the severity of the burn trauma and influencing the evolution of the patient.

The ABSI score is a five-variable scale used as a prognostic factor in evaluating the mortality risks of burn patients worldwide for a long time. It incorporates age, sex, (TBSA burned, presence of full-thickness burns, and inhalation injury to help estimate patient outcomes and expected length of hospital stay. In our cohort, the mean ABSI score was 7.31 ± 3.09, consistent with other reports, although many studies document lower average values. The ABSI score classifies a value of 7 as moderately severe, with an 80-90% probability of survival. In our study, 50% of patients had scores ≤7, while 25% had scores ≥9. The mean ABSI among survivors was 6.11 ± 2.00, significantly lower than in deceased patients, with a mean score of 11.01 ± 2.54. Moreover, ABSI values >13 were associated with a marked reduction in LOS. ABSI score was a statistically positive predictor (B = 0.95; SE = 0.22; Wald_(df=1)_ = 19.23; *P* < 0.001) with each unit increase corresponding to a 2.6-fold increase in the risk of death (OR = 2.58; 95% CI, 1.69–3.93) [14-16].

With the observed reduction in burn-related mortality over recent decades, LOS has increasingly been adopted as a key outcome measure. Various studies have attempted to identify factors, such as socioeconomic status, that may influence LOS in burn patients. [17]. Estimating LOS at the time of admission can help clinicians set discharge goals, plan resource allocation, and assist families in preparing for the patient’s recovery trajectory. It is commonly estimated as one day for each percent of the burned surface, although the LOS frequently exceeds this estimation. Two apparent exceptions that do not adhere to this rule are the patients who have a high burned TBSA or those with inhalation injuries due to greater physiological stress and higher complication rates, especially infections [18]. Most studies report mean LOS values between 8 and 14 days, while the current study presents a significantly higher value, ranging from 1 to 131 days with an asymmetrical positive distribution (skewness = 1.90; kurtosis = 3.78) having an important presence in the lower interval of values and some extreme values in the superior aspect with a mean length of stay of 24 days (23.42 ± 23.47) and a median value of 18 days, 25% of patients being admitted for more than 30 days into the hospital [19,20].

The overall mortality rate in our cohort was 28.5%, which is higher than that reported in many other studies, reflecting the severity of the burn trauma and correlating with the high ABSI score. Mortality in burn patients is multifactorial and influenced by all five components of the ABSI score. Consistent with previous literature, our data showed an 11-fold increase in mortality among patients older than 55 years (*P* < 0.001) and a 9-fold increase associated with inhalation injuries (*P* < 0.001). Among functional regions, the most substantial impact on mortality was observed in patients with perineal burns, which increased mortality risk sixfold, followed by facial burns, associated with a threefold increase [21]. Other statistically significant predictors of mortality described in the literature include male sex, TBSA burned, TBSA grafted, and comorbidities [22].

Although TBSA burned remains one of the primary metrics for assessing burn severity, it does not adequately capture the functional consequences associated with injuries to critical anatomical regions. An alternative method is using the cutaneous functional units (CFU), which are skin segments that accommodate the normal range of movement and may become contracted following the injury, thus becoming a more accurate indicator of the severity of the burn by measuring the functional consequences. Even though the hands account for only 5% of the TBSA, they represent 81.3% of the body’s CFU. As a result, burn injuries involving the hands are disproportionately associated with poorer physical outcomes, including contractures, deformities, loss of function, occupational limitations, and psychosocial distress. Thus, recent studies advocate for the clinical superiority of CFU-based assessment, particularly in settings where individualized functional rehabilitation is prioritized [23,24].

Hand function is essential for everyday activities, and even relatively minor burns can severely impair functionality, either due to the injury itself or from resulting scar contractures. Preserving and restoring hand function is critical for the long-term well-being of burn survivors, as impairment can lead to social withdrawal and long-term disability. Despite representing only 3% of the TBSA, they always have a primary indication for treatment. Studies report a more than 80% involvement in all severe burns, making them one of the most affected parts. Despite having a lower incidence in this study (63.6%) than in the literature, the frequency is still very high [25]. Recognizing the depth of the burn requires experience and practice, which are paramount to formulating an appropriate treatment plan that includes a meticulous surgical technique and preoperative and postoperative hand therapy to maximize functional recovery [26].

The depth, size, and healing time of a burn injury are key factors in determining the need for surgical intervention. Superficial second-degree burns typically heal by secondary intention with topical antimicrobials and appropriate dressings, thereby avoiding the additional scarring often associated with skin grafting. In contrast, deep partial-thickness burns may require surgical excision if they do not heal within 2 weeks, while full-thickness burns almost always necessitate excision followed by skin grafting. A relative contraindication to early excision in hand burns may arise in patients with extensive TBSA involvement. Patient stabilization and grafting of larger, life-threatening areas become a priority in such cases. Once those sites are healed, grafts can be harvested to cover the hands. During this period, temporary treatments, such as enzymatic debridement, allografts, dermal substitutes, or cultured epithelial cells, can manage hand burns until definitive coverage. Current evidence does not report any notable difference in the outcome when comparing late versus early excision in the burned hands, provided that the therapy is performed continuously by maintaining the range of motion (ROM) of the wrist and the fingers and using orthosis [26].

The best results are obtained when planned rehabilitation is performed with intensive physical and occupational therapy. Active movement should be promoted from the initial stages to prevent contracture formation. If these appear, they should be released and splinted in the ideal positions or used in dynamic splints, encouraging active motion after surgical corrections. Scar management is equally critical to functional rehabilitation, notably through custom-fitted pressure garments, which are typically initiated two weeks post-grafting. These garments are worn for up to 23 hours per day for 6 months in cases involving deeper burns or prolonged healing. The continued use of pressure garments depends on the individual response and scar characteristics, with improvements noted in scar color and texture over time. For burns involving web spaces, specialized spacers may be required to prevent contracture and maintain anatomical contours. Silicone sheets have also been proven useful, especially on the dorsal part of the finger and the web spaces when placed beneath the pressure garments. Hand therapy should be continued until the function returns to normal or a plateau is noted in improving movement. A maintenance program may be useful with or without further surgical procedures [27].

Burns on the face are especially disabling due to the involvement of highly specialized and anatomically complex structures, such as the eyes, nose, mouth, and ears, and may extend into the cervical region. In severe cases, such injuries can result in facial stigmata. Management of facial burns must address two equally important aspects: functional restoration (e.g., breathing, eating, communication) and esthetic rehabilitation, as the face plays a central role in identity and social interaction. Scars should ideally be unobtrusive at conversational distance. The impact of facial burns on self-image and social functioning often leads to a significantly reduced quality of life. Burns on the face require prompt treatment from the healthcare team to alleviate mortality and morbidity. In developed countries, burn mortality has continuously declined over the past decades; thus, optimal management and reconstruction are important to improve function, esthetic outcomes, and quality of life [28]. Of the 319 patients who had burns on the cephalic extremity, 313 had facial burns, representing 65% of the total 481 patients, being a positive marginal predictor (B = 1.00; SE = 0.54; Wald_(df=1)_ = 3.43; *P* = 0.064) with a 3- fold increase in morbidity (OR = 2.71; 95% CI, 0.94–7.81).

The perineum is a particular anatomical region frequently exposed to contamination, which may lead to sepsis, graft loss, delayed wound healing, and abnormal scar formation [29]. The association with urinary tract infections, risk of fecal contamination, increased incidence of infections acquired in the hospital, and bacteriemia has an important impact on the quality of life, as well as higher morbidity and mortality. [1,10,30,31]. Due to its anatomical protection and specific skin characteristics, perineal burns rarely occur in isolation. In most cases, they are associated with thigh burns as well as the anterior abdominal region and are usually seen in patients with extensive TBSA involvement, ranging from 21% to 56% [32]. In our study, 86 patients (17.9% of the cohort) sustained perineal burns, which were associated with a significantly higher risk of mortality (B = 1.81; SE = 0.50; Wald_(df=1)_ = 13.21; *P* < 0.001), corresponding to a sixfold increase in the likelihood of death (OR = 6.13; CI 95%, 2.31–16.31) compared to patients without perineal involvement.

As burn survival rates improve, greater emphasis has been placed on minimizing post-burn morbidity and optimizing functional and aesthetic outcomes. One major source of post-injury disability is contracture formation. Although the exact incidence and associated risk factors remain unclear, contractures remain a significant complication, with some studies reporting that 33% of patients develop at least one contracture by discharge. The most frequently affected joints include the shoulder (23%), elbow (19.9%), wrist (17.3%), ankle (13.6%), and knee (13.4%). Reported facial contractures include microstomia (0.27%), ectropion (0.86%), and nasolabial contractures (0.16%). The majority of contractures were classified as mild (47.2%) or moderate (32.9%) [22].

The high incidence of contracture could be explained by multiple reasons: regional centers often treat the most serious injuries, the critical ones, with a higher TBSA burned grafted, thus having a higher risk of forming contractures. Larger burns usually cross multiple joints and require more interventions, leading to a lengthier immobilization and a higher contracture rate. Larger burns also require an extended ICU stay, which, by itself, if longer than 14 days, may lead to an important rise in contracture rates. Thus, the overall contracture incidence is directly related to the examined number of joints or burn locations [22,33].

A common pathological evolution following burn injuries is the formation of scar contractures, which limit the range of motion and impair the function of the affected joints. Thus, the use of static splinting has long become a preventative practice. The most frequently splinted joints were the elbow, the wrist, the knee, and the ankle. Studies report more frequent use of splints in larger burns and those with increased severity that had a higher need for grafting in the CFU of the joints (with a six-time increase of odds). The wrist was the most frequently splinted joint in approximately 30.7% of cases, while the knee was the least (8.2%). About one-third of patients continued splint use until hospital discharge [34].

Although the true prevalence of contractures following burn injury is inconsistently reported, with considerable variability across studies, a comprehensive review by Oosterwijk *et al*. provides several valuable insights [35]. Their findings indicate a high incidence of scar contractures at discharge, ranging between 38% and 54%, though the prevalence tends to decrease over time. The study identified several risk factors for contracture formation, including deep burns, surgically treated wounds, and involvement of specific anatomical joints. Additionally, female patients and children were found to be at higher risk. The study identified several risk factors for contracture formation, including deep burns, surgically treated wounds, and involvement of specific anatomical joints. Additionally, female patients and children were found to be at higher risk [35]. The neck and the upper extremities are more prone to developing contractures than the lower extremities, and the vast majority (80%) of these cases necessitated surgery in previously skin-grafted areas. In addition, the duration of splinting may influence scar contractures, with a high prevalence (over 90%) in patients who did not receive splints or were splinted for less than 6 months compared to the group with splinting for more than 6 months, noting a prevalence of 17%. Thus, there is a strong consensus on the need for further research into the effects of splinting in scar contracture and a better understanding of the effectiveness they have on myofibroblast activity and scar maturation, more so because contracture severity classifications are described but not related to the overall function [36-38].

Our center serves as a national referral facility, receiving a high volume of complex burn cases, including patients with severe injuries and those with associated trauma. A notable proportion of these patients (51.1%) originated from rural areas, where limited access to specialized burn care and health illiteracy contribute to significant delays in presentation. These delays reduce the opportunity to implement early interventions to prevent complications such as contractures. Although numerous studies have addressed the challenges of burn care, there remains a lack of a standardized classification protocol for functional anatomical regions adaptable to the specific needs of different countries and burn centers.

Of the 481 patients in our study, 293 (61%) required surgical intervention. Among these, 249 patients (85%) had involvement of at least one functional anatomical area, whereas only 44 patients (15%) underwent surgery without such involvement. Further analysis of surgical procedures revealed that the hands were the most commonly operated region, with 198 surgical interventions, followed by other major joints: 93 elbows, 81 knees, 61 feet, and 43 shoulders. The anatomical regions that required fewer operations were the hips, followed by the cervical region and the face, as well as the perineal region, which were treated mainly by conservative measures. Given the high TBSA affected and the considerable depth of burn injuries in many cases, there was a need for urgent surgical coverage of extensive areas. Consequently, split-thickness skin grafts were the most commonly performed procedure in 227 cases. Full-thickness grafts were used in only 12 patients, flap reconstructions in 19, and direct sutures in 38 cases.

This burn unit forms an integral part of the largest emergency hospital in Bucharest, receiving many patients from across the country. As such, most patients are treated here, and this protocol mostly addresses the acute phase of the burn treatment. In cases of burn centers such as ours, where most patients are severe with a high burned TBSA and deep burns, the most important issue is to cover the large burned surfaces to limit the liquid and protein losses. Early excision and grafting are employed to reduce metabolic stress and systemic inflammation, thus stabilizing the patient, particularly in more vulnerable individuals, such as the elderly or those with comorbidities. This approach is applied in both full-thickness (third-degree) and deep partial-thickness burns, using split-thickness skin grafts harvested from uninjured areas. Typically, up to 15% TBSA is grafted per session weekly and tailored to the patient’s lab results. Temporary skin substitutes or cadaveric allografts may facilitate initial wound coverage and promote stabilization. On the other hand, full-thickness skin grafts are usually reserved for the more sensitive areas, such as the face and the hands, which are rather scarce resources.

Patients requiring rapid coverage of extensive burns are also those at the highest risk of mortality. We found the patients with the highest risk to be those >55 years old, those with inhalation injuries, or those with a high ABSI score. In our cohort, 55.9% had full-thickness burns, and 90% of these were also associated with deep partial burns, leading to an increase in the overall deep burns and an increase in the severity of the patients, requiring even more skin grafts than other patients.

After achieving coverage of extensive burn areas and stabilizing the patient, attention should shift to functional anatomical regions, prioritizing those associated with a higher risk of mortality or those most frequently affected. In our study, the face was among the earliest treated regions, as facial burns were linked to a threefold increase in mortality risk. Additionally, facial burns were frequently associated with hand burns, observed in 77.5% of cases. Due to their anatomical proximity, burns of the cephalic extremity were commonly accompanied by injuries to the shoulders (87.2%) and elbows (84.1%), which were often managed concurrently. Other joints, such as the knees, although involved in 53.3% of cases, were less frequently met. As a result, these areas were typically addressed in subsequent phases of treatment, as no direct correlation was found between their involvement and worsened clinical outcomes. One particularly critical functional area is the perineum, which demonstrated a six-fold increase in mortality risk in our study. While most perineal burns are superficial and can be managed conservatively, deeper injuries in this region may necessitate surgical intervention.

An alternative approach to the treatment protocol, prioritizing functional areas first, is typically reserved for patients with smaller TBSA involvement, fewer grafting requirements, and a more stable general condition. As stated before, we usually treat functional areas with full-thickness or thicker split-thickness skin grafts. Because donor sites for these grafts are limited, particularly for full-thickness grafts, it is essential to allocate these resources judiciously and ensure the highest likelihood of successful graft take. To optimize graft success, preoperative laboratory parameters must indicate favorable conditions, including adequate hemoglobin levels, total protein, an appropriate albumin-to-globulin ratio, and the absence of wound contamination and strong patient compliance. A successful graft take is associated with reduced immobilization time, faster recovery, and improved functional and aesthetic outcomes, ultimately minimizing long-term sequelae.

## CONCLUSION

The subject of the order to treat burns of different anatomical regions remains a topic of great debate. Some authors recommend treating the functional areas first, as they are more 'precious' and should be prioritized, while others advocate for covering the larger surfaces first to limit fluid and protein losses and stabilize the patient, treating the functional areas only afterward. Whichever protocol is chosen, it should be adapted to the specific characteristics and needs of each burn center, which may vary from one institution to another. As a general rule, covering large burned surfaces and stabilizing the patient should be the initial priority. Functional areas can then be treated, often with full-thickness skin grafts, frequently requiring further operations to improve the functional outcome, but only once the patient is stabilized and in good general condition.
